# A versatile computational algorithm for time-series data analysis and machine-learning models

**DOI:** 10.1038/s41531-021-00240-4

**Published:** 2021-11-09

**Authors:** Taylor Chomiak, Neilen P. Rasiah, Leonardo A. Molina, Bin Hu, Jaideep S. Bains, Tamás Füzesi

**Affiliations:** 1 Division of Translational Neuroscience, Department of Clinical Neurosciences, Hotchkiss Brain Institute, Alberta Children’s Hospital Research Institute, Cumming School of Medicine, University of Calgary, 3330 Hospital Drive, Calgary, AB T2N 4N1 Canada; 2 CSM Optogenetics Facility, University of Calgary, 3330 Hospital Drive, Calgary, AB T2N 4N1 Canada; 3 Department of Physiology & Pharmacology, Cumming School of Medicine, University of Calgary, 3330 Hospital Drive NW, Calgary, AB T2N 4N1 Canada

**Keywords:** Computational neuroscience, Predictive medicine

## Abstract

Here we introduce Local Topological Recurrence Analysis (LoTRA), a simple computational approach for analyzing time-series data. Its versatility is elucidated using simulated data, Parkinsonian gait, and in vivo brain dynamics. We also show that this algorithm can be used to build a remarkably simple machine-learning model capable of outperforming deep-learning models in detecting Parkinson’s disease from a single digital handwriting test.

Dynamic biological signals are comprised of subtle underlying features that may be hidden from commonly used analysis tools. The advent and rapid adoption of analysis approaches has increased our ability to identify recurring patterns within these signals with important implications for the identification of diagnostic biomarkers^1–6^. While the most well-known methods for time-series data analysis are those based on linear concepts such as correlation and power spectra^2,3^, nonlinear tools can extract important information associated with underlying signal patterns that may not be fully captured with linear methods^3,4^. One challenge is that traditional nonlinear approaches such as recurrence analysis require thresholding^2^, meaning that exclusion of a large proportion of the data may result in loss of potentially important signal information.

The affordability of portable technology has enabled the quantitative assessment of dynamic signal data for Parkinson’s disease (PD) on a widespread basis, often without the need for expensive equipment or dedicated space^7^. For example, digital handwriting analysis has been shown to be a successful diagnostic strategy for PD and is more efficient compared to paper handwriting tests as well as costly and time-consuming expert assessment, neurological tests, and/or brain imaging^8^. While deep-learning (DL) techniques have been developed using raw images^9^, recurrence analysis^5^, or spectral analysis^6^ as input features to detect PD from digitized tablet handwriting samples, these are limited by the added computational complexity associated with the deep architecture and the need for large datasets for training which can have an impact on other clinical decision-support system-level constraints such as cost, computational time, and budget^8,10,11^.

Here we present Local Topological Recurrence Analysis (LoTRA), a simple yet versatile analysis method that helps overcome these limitations that can be used to reveal highly discriminant recurring patterns embedded in *n*-dimensional time-series data.

To illustrate the general concept of LoTRA, three phase-space trajectories were created from a simple periodic signal (sine wave) by changing the 2-dimensional embedding delay, thus creating three different sine wave phase-space trajectories (data clouds) with varying topological shape that can be distinguished by LoTRA (Fig. 1a–c). The blue and black ribboning in the LoTRA plots correspond to the shallow curvature of the phase-space trajectory and the directionality of the trajectory; while the repeating red and green pattering correspond to the increased curvature associated with the vertices along the trajectory path (Fig. 1b–c; also see “Methods”). In fact, LoTRA was able to capture unique multi-dimensional signal information that may be missed by traditional recurrence or spectral analysis (Supplementary Figs. 1–3).

**Fig. 1:**
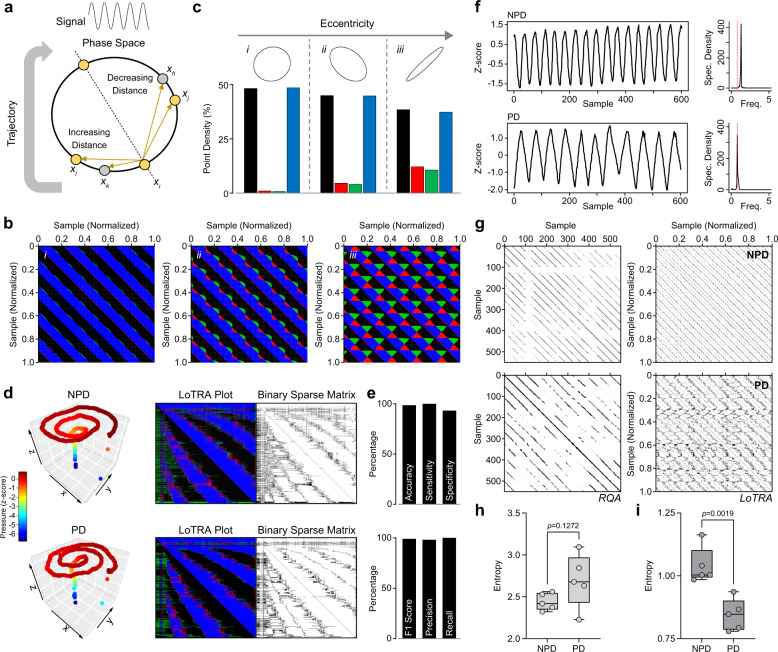
LoTRA: Parkinson’s gait and handwriting. **a** Top, 1-dimensional dynamic signal; sine wave. Bottom, 2-dimensional phase-space reconstruction of the sine wave signal where it is easy to distinguish between *x*_*i*_−*x*_*l*_ and *x*_*i*_−*x*_*j*_ based on local neighborhoods. **b** LoTRA plots of three different phase-space reconstructions (inset in **c**). **c** Colors represent quartiles of the 8-bit binary code associated with topological features used to create a binary matrix to parse the signal’s component parts (see “Methods”). **d** Multi-dimensional time-series data for digital handwriting samples from the Dynamic Archimedean Spiral Test for non-PD (NPD) and PD (left), their associated LoTRA plots (center), and their high-curvature binary sparse matrices (right) with which recurrence statistics are extracted. **e** Top; diagnostic-based performance metrics: accuracy = 98.6%, sensitivity = 100%, and specificity = 93.3%. Bottom; classification model performance metrics: F1 score = 99%, precision = 98.2%, and recall = 100%. **f** NPD and PD gait data from a single wearable sensor (left). Shifted spectral peak in PD indicting slower gait cycling (right). **g** Additional information related to underlying disturbances in gait-cycle complexity captured by recurrence plots (left) and LoTRA plots (right) for each trace shown. **h** RQA summary data (*t*_(8)_ = 1.702, *p* = 0.127). **i** LoTRA summary data (*t*_(8)_ = −4.542, *p* = 0.002). All box plots represent median and upper and lower quartiles.

Next, we used data collected from a handwriting test administered to individuals with and without PD to build a simple machine-learning model and evaluate if LoTRA features can translate into effective feature-space discriminability. Data from a single Dynamic Archimedean Spiral Test captured on a graphics tablet were used^8,12^ which has participants trace an Archimedean spiral with a stylus as the spiral blinks^12^. This prevents participants from relying on visual cues to simply trace the spiral while continuing to draw, challenging both working memory and motor ability^9,12^. Multi-dimensional traces consisting of the *x*–*y* coordinates and corresponding pressure applied by the stylus over time are shown in Fig. 1d (left), while the LoTRA-generated binary sparse matrices that were used to extract a short (length = 9) feature vector^13^ for support vector machine classification are shown in Fig. 1d (right). Model performance metrics obtained by nested leave-one-out cross validation are shown in Fig. 1e. Using only a single test and a feature vector a fraction of the length, LoTRA can be used to build a remarkably simple machine-learning model with improved performance compared to other state-of-the-art DL achievements on this dataset^5,6,9,14^ (Fig. 1e; Supplementary Table 1). These results are also comparable to other DL-based models requiring more trials, tests, and/or samples^8,15^. As the feasibility of telehealth/telemonitoring is heavily dependent on the speed and quality of the internet connection^16^, algorithms that are highly effective while requiring fewer tests/less data would be highly advantageous in both helping to develop more efficient and accessible telehealth/telemonitoring systems (particularly in rural areas), and for conserving battery life on portable devices.

While gait speed has become the standard gait parameter in PD because it is easy to measure and interpret, reduced gait speed alone might not be suitable to characterize the disease course^17^. Rather, identification of underlying disturbances in gait-cycle fluidity may manifest as changes in complexity (entropy) and provide a better indication of locomotor control system breakdown^1,17^. In fact, we found that LoTRA may represent a powerful tool for revealing underlying disturbances in the complexity of PD gait cycling compared to traditional recurrence analysis (Fig. 1f–i).

Finally, our proposed algorithm may have a number of applications related to neurology/neuroscience in general, and may be particularly useful for studying stress and anxiety in animal models of PD^18^. For example, computational simulations have shown that increased underlying neural ensemble phase locking and coherence can result in decreased complexity of the overall population-level dynamic signal of a network^19^. Indeed, using a genetically encoded fluorescent calcium reporter (GCaMP6f) in stress-related neurons of the hypothalamic paraventricular nucleus (Fig. 2a, b), LoTRA was found to support these simulation studies as exposure to a mild stress (a novel environment) was associated with reduced population-level signal complexity and increased underlying correlated neuronal activity patterns (Fig. 2c–g).

**Fig. 2:**
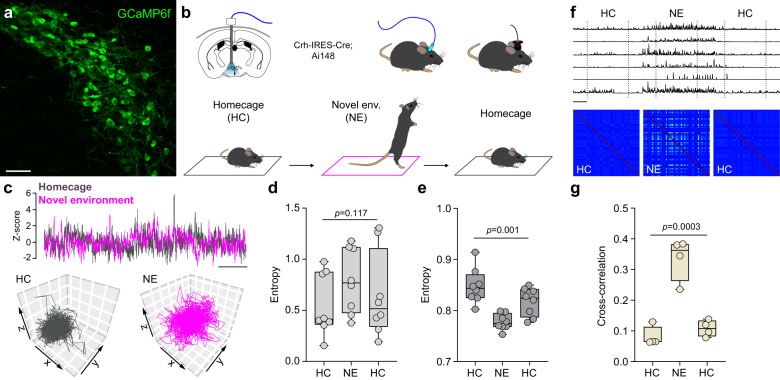
LoTRA and in vivo brain dynamics. **a** Expression of GCaMP6f (green) in the paraventricular nucleus corticotropin-releasing hormone (PVN CRH) synthesizing neurons. **b** Schematic of the experimental design and exposure to a mild stress (novel environment). **c** Example GCaMP6f recordings (top) and 3-D plots of the reconstructed fiber photometry population-level signals (bottom). **d** Summary RQA data (repeated-measures ANOVA *F*_(2,14)_ = 2.514, *p* = 0.117; Bonferroni correction *p* ≥ 0.104). **e** Summary LoTRA data (repeated-measures ANOVA *F*_(2,14)_ = 11.741, *p* = 0.001; Bonferroni correction *p* ≤ 0.007 compared to NE). **f** Sample traces of individually identified units from one animal for each condition (top) and cross-correlation matrices (bottom). **g** Summary data between the activity of PVN CRH neurons in the three conditions (HC: 0.08 ± 0.02, NE: 0.34 ± 0.03, HC: 0.11 ± 0.01; HC/NE: *p* = 0.0005; HC/HC: *p* > 0.9999; NE/HC: *p* = 0.0009; *n* = 4; repeated-measures ANOVA *F*_(2,6)_ = 41.33, *p* = 0.0003, Bonferroni correction). Scale bars: **a**, 50 µm; **c**, 25s; **f**, 60s.

Taken together, our proposed algorithm provides an opportunity for more elaborate analysis and the extraction of richer data from a variety of time-series data types.

## Methods

### Ethics approval

All animal protocols were approved by the University of Calgary Animal Care and Use Committee. Human data used in this study were collected in previous studies with informed consent and ethics approval by the ethics committee of Cerrahpaşa Medical Faculty of Istanbul University^12^, as well as the University of Calgary University Ethics Board for Human Research and the Committee of the First Affiliated Hospital of Sun Yat-sen University^20^.

### Theoretical framework

An important strategy for gaining deeper insight into underlying dynamic changes obtained by data series is to extract recurring patterns embedded within these data through distance-based thresholding and recurrence quantification analysis (RQA)^1,20–30^. However, choosing the threshold parameter setting to define what is “recurrent” is not always an easy task^31^. In a dynamic state, a recurrence has occurred if at time *t*_*j*_, a trajectory of a system returns into the dynamical neighborhood of a previous state $x_i = x(t_i)$, where $t_i \,<\, t_j$^28,32^. Such neighborhoods commonly rely on distance metrics to quantify proximity, and thus recurrent patterns, by applying a distance-based threshold to a Heaviside step function^28,30^. This creates a binary recurrence matrix which can be easily visualized via the recurrence plot^20,28,30,33,34^. These matrices are, however, often not transitive; that is, $R_{ij} = 1$ and $R_{jk} = 1$ does not necessarily imply $R_{ik} = 1$^28^. This may limit the identification of recurrent patterns and emergent recurring domains^28^. That is because with thresholding important information related to underlying dynamic processes embedded within the signal may be lost, or spurious patterns introduced with inappropriate thresholding^28,30,31,35^.

Consider for a moment the traditional recurrence analysis approach for time-series data:

1 $${{{\mathbf{x}}}} = (x_1,x_2,x_3, \ldots ,x_n)$$

where ${{{\mathbf{x}}}}$ represents a time-series vector. If only a one-dimensional time-series vector is available (as above), time-delay embedding can be used to reconstruct the phase-space trajectory for the time-series, where *m* is the embedding dimension and *t* is the embedding delay:

2 $$V = \left( {\begin{array}{*{20}{c}} {\begin{array}{*{20}{c}} {V_1} \\ {V_2} \end{array}} \\ {V_3} \\ \vdots \\ {V_{n - (m - 1)t}} \end{array}} \right) = \left( {\begin{array}{*{20}{c}} {x_1} & {x_{1 + t}} & { \ldots x_{1 + \left( {m - 1} \right)t}} \\ {\begin{array}{*{20}{c}} {x_2} \\ {x_3} \\ \vdots \end{array}} & {\begin{array}{*{20}{c}} {x_{2 + t}} \\ {x_{3 + t}} \\ \vdots \end{array}} & {\begin{array}{*{20}{c}} { \ldots x_{2 + \left( {m - 1} \right)t}} \\ { \ldots x_{3 + \left( {m - 1} \right)t}} \\ \vdots \end{array}} \\ {x_{n - \left( {m - 1} \right)t}} & {x_{n - \left( {m - 2} \right)t} \ldots } & {x_n} \end{array}} \right)$$

with the elements of *V* all being constructed from ${{{\mathbf{x}}}}$ based on some delay, *t*. The delay, like the embedding dimension, *m*, is estimated quantitatively^1,20,24,36^. The recurrence matrix $R = R_{ij}$ is 1 if *x*_*j*_ is contained in a “ball” $B_\varepsilon (x_i)$ of radius $\varepsilon$ > 0, and 0 otherwise as mediated by applying a Heaviside step function to the distance matrix to generate the recurrence plot^28,33,34,37^:

3 $$R_{ij} = {{\Theta }}(\varepsilon - \parallel {{{\mathrm{V}}}}_i\left( {{{\mathbf{x}}}} \right) - {{{\mathrm{V}}}}_j({{{\mathbf{x}}}})\parallel )$$

where ${{\Theta }}$ is the step function which is 0 for values <0 and 1 for values ≥0, and $\varepsilon$ represents the threshold parameter^23,36^. However, setting up an optimal $\varepsilon$ can be challenging, and we are also limited by the fact that significant dynamic features may be lost by $\varepsilon$-thresholding^31,35,38^. With thresholding distance we are only including points along the trajectory that are “close” together without considering the context of the local neighborhood. This means that recurring patterns with respect to changes in phase-space trajectories are indistinguishable when $x_j$ is contained within the ball of radius $\varepsilon$, and are excluding altogether when $x_j$ is outside the ball of radius $\varepsilon$.

Now consider the 3x3 neighborhood around each vector element being constructed from ${{{\mathbf{x}}}}$. ${{{\mathbf{x}}}}$ represents our one-dimensional time-series vector that can be used for time-delay embedding with delay *t* and embedding dimension *m* to reconstruct the phase-space trajectory^1,20,36^. However, instead of considering the Euclidean norm between data points:

4 $$D_{ij} = (\parallel {{{\mathrm{V}}}}_i\left( {{{\mathbf{x}}}} \right) - {{{\mathrm{V}}}}_j({{{\mathbf{x}}}})\parallel )$$

we will consider vectors *V*_*i*_ and *V*_*j*_ in relation to their local 3x3 neighborhood such that:

5 $$T_{ij} = \left( {\begin{array}{*{20}{c}} {D_{i - 1j - 1}} & {D_{i - 1j}} & {D_{i - 1j + 1}} \\ {D_{ij - 1}} & {D_{ij}} & {D_{ij + 1}} \\ {D_{i + 1j - 1}} & {D_{i + 1j}} & {D_{i + 1j + 1}} \end{array}} \right)$$

where it can be seen that $T_{ij}$ ≠ $T_{il}$ even when $D_{ij}$ = $D_{il}$ (Fig. 1a). It follows then, that directional and curvature information can be captured by different inequality patterning around the 3x3 neighborhood when computed for all $T_{ij}$ by constructing a new matrix that represents an 8-bit binary code for each point-pair’s local neighborhood $\left( {T_{ij}^\prime } \right)$:

6 $$\left( {T_{ij}^\prime } \right)_8 = \mathop {\sum }\limits_{n = 1}^8 s\left( {g_n - g_0} \right)2^{n - 1};s\left( x \right) = \left\{ {\begin{array}{*{20}{c}} {0,\;x \,<\, 0} \\ {1,x \ge 0} \end{array}} \right.$$

where *g*_*0*_ represents $(D_{ij})$ and *g*_*n*_ = {*g*_*1*_, … ,*g*_*8*_} are its eight connected neighbors^39,40^. Each neighbor which is larger or equal to *g*_*0*_ is set to 1, while each neighbor that is smaller than *g*_*0*_ is set to 0. A binary code is thus created by moving around the central point *g*_*0*_ (here counterclockwise) where a single integer value is calculated based on the sum of the binary code elements (0 or 1) multiplied by the eight 2^p^ positional weights (increments of powers of 2: 2^0^, 2^1^, 2^2^, 2^3^, 2^4^, 2^5^, 2^6^, 2^7^) starting with the preceding points (i.e., $D_{i - 1j - 1}$). This represents 8-bit binary coding where there are 2^8^ (256) different possible integer values, ranging from 0 to 255. This newly created matrix now allows for the identification of graded changes in phase-space trajectories that can be used to identify recurring sets of local topological features embedded within the dynamic signal (LoTRA plot; see Fig. 1b, c for example).

To illustrate the basic idea of LoTRA, three phase-space trajectories were created from a simple periodic system (sine wave) by changing the 2-dimensional embedding delay (see next section), thus creating three different sine wave phase-space trajectory topologies (or data clouds) with increasing eccentricity (Supplementary Fig. 1; left, top to bottom). The recurrence plots from traditional recurrence analysis for each are also shown (Supplementary Fig. 1; middle, top to bottom), illustrating the characteristic diagonal line patterning of periodic systems. The varicosities associated with the highly eccentric structure (Supplementary Fig. 1; middle, bottom panel) illustrate how distance-based thresholding can include the unwanted parallel trajectory segments, running in opposite time, as “recurring” points^31^. Alternatively, the LoTRA plot can be used to characterize and compare the different features associated with the data cloud in phase-space (Supplementary Fig. 1; right, top to bottom). The blue and black ribboning correspond to the shallow curvature of the phase-space trajectory and the directionality of the trajectory; while the repeating red and green pattering correspond to the increased curvature associated with the vertices along the trajectory path with changing eccentricity of the different structures relative to point $x_i$ along the line of identity. When considering the right panel, moving from top to bottom, it becomes apparent that LoTRA indeed captures additional information related to this signal’s phase-space trajectory that is lost by thresholding with traditional recurrence analysis (Supplementary Fig. 1; compare middle and right panels).

We also considered a more complex simulated system; the Lorenz attractor^28,30^. This system is widely used for the study of dynamic processes as it exhibits characteristic oscillatory dynamics and recurring domains^28,41^. Supplementary Fig. 2a, b display the 3-dimensional time-series data of the Lorenz attractor. The recurrence plot in Supplementary Fig. 2c exhibits the typical diagonal line patterning that is characteristic for oscillatory dynamics and the “wings” of the Lorenz system that are explored by the system’s trajectory^28^. The results of LoTRA for the same dynamic states are shown in Supplementary Fig. 2d, e. Similar to the periodic sine wave signal, additional dynamic signal information can be captured by LoTRA.

### Simulated data

For an idealized periodic signal, a simple sine wave was constructed by a sequence of 300 points, ranging from 0.1 to 30 with an interval of 0.1. To modify the eccentricity of the *m-*dimensional phase-space trajectory of the sine wave when *m* = 2, the embedding-delay (*t*) was changed for phase-space reconstruction. Values of *t* = 15, *t* = 20, and *t* = 3 were used, where *m* is the embedding dimension and *t* is the embedding delay with the elements of *V* all being constructed from ${{{\mathbf{x}}}}$^1,20,36,42^. The distance matrix was generated using the Euclidean norm. For a more complex simulated dynamic system, the Lorenz attractor was used^28,41^. For this study, simulation of the data series was initialized with parameters *r* = 28, *σ* = 15, $\beta$ = 8/3, and ∆*t* = 0.025.

### Handwriting dataset

Data and specific dataset details for the Dynamic Spiral Test (Parkinson Disease Spiral Drawings Using Digitized Graphics Tablet Data Set)^9,12^ for control and PD are publicly available at the UC Irvine Machine Learning Repository (https://archive.ics.uci.edu/ml/index.php). The Handwriting dataset was constructed using a Wacom Cintiq 12WX graphics Tablet^9,12^. These data are already multi-dimensional (*x*–*y* coordinates and pressure) and thus time-delay embedding was not used. As not all data were acquired at the same sample rate^6^, the data were downsampled by every three or four points so all data had approximately equal sampling rates.

### Human gait data

Human gait data were previously collected using the Ambulosono wearable system^20^. This system utilizes high-precision movement sensors consisting of a 3-axis Micro-Electro-Mechanical Systems (MEMS)-based gyroscope and a 3-axis accelerometer. The firmware uses proprietary fusion codes for automatic gravity and sensitivity calibration and real-time attitude angles output (pitch, roll, and yaw), while the GaitReminder App utilizes algorithms to sample sensor output for real-time gait calculations after corrections for limb length, angular excursion, signal filtering and drift^43,44^. The data series consisted of 12 sec of gait-cycling data sampled at 50 Hz from *n* = 10 subjects, with half the sample having been diagnosed with mild to moderate Parkinson’s disease (PD) with gait disturbances, and the other half of the sample corresponding to neuro-typical individuals without any neurological or gait impairments^20^. For both LoTRA and traditional recurrence quantification analysis, the data series were first reconstructed in *m*-dimensional phase-space by estimating the delay time *t* and the embedding dimension *m*. Here, the delay time *t* (range 9–19) and the embedding dimension *m* (range 2–6) for each z-score scaled data series analyzed were determined using Average Mutual Information (AMI) and the False Nearest Neighbor (FNN) functions, where the first local minima of those functions or the point at which those functions level-off or no longer appreciably change can be used to estimate the delay and embedding dimension^45^. For RQA the same *t* and *m* for each time-series were used as in LoTRA, but with the additional threshold parameter adjusted to generate a recurrence rate on the order of 4–5%. This recurrence rate is consistent with the estimated optimal value which has been used previously for periodic gait^1,46^.

### Mice

For the experiments, the offspring of *Crh-IRES-Cre (B6(Cg)-Crhtm1(cre)Zjh/J*; stock number 012704) mice crossed with *Ai148 (Ai148(TIT2L-GC6f-ICL-tTA2)-D*; stock number 030328) animals were utilized. Mice were obtained from Jackson Laboratories. Mice were housed on a 12-h:12-h light:dark cycle (lights on at 7:00 a.m.) with ad libitum access to food and water in whole litters until 1–2 d before use, then were individually housed during the experimental phase. Mice were 6–8 weeks old at the time of ferrule or lens implantation.

### GRIN lens and fiber implantation

*Crh-IRES-Cre;Ai148* mice were maintained under isoflurane anesthesia in the stereotaxic apparatus. For fiber photometry, a 400 µm diameter mono fiber optic cannula (Doric Lenses, MFC_400/430/0.48_5mm_MF2.5_FLT) was implanted dorsal to the PVN (AP, −0.7 mm; L, −0.2 mm from the bregma; DV, −4.5 mm from the dura. The GRIN lens (7.3 mm length; Inscopix) was lowered dorsal to the PVN at a 100 µm/min speed using a motorized stereotaxic apparatus. The implantations were targeted to the PVN and were affixed to the skull with METABOND® and dental cement. At least one month after lens implantation a baseplate was installed on the head. Experiments started after an additional two weeks of recovery and handling.

### In vivo fiber photometry and miniature microscope data

Fiber photometry was used to record GCaMP6f (*n* = 8, *Crh-IRES-Cre;Ai148*) calcium transients from CRH neurons in the PVN of freely moving mice. Animals were handled for 5 min a day for 3 successive days and then habituated to the optic fiber in their homecage (15 min a day) for 3 additional days. We recorded 10 min of PVN CRH neuron activity in the homecage immediately before and after each test for better bleaching correction. A fiber photometry system was used from Doric Lenses: Consisting of two excitation LEDs (465 nm and 405 nm from Doric) controlled by a LED driver and console, running Doric Studio software. The LEDs were modulated at 208.616 Hz (465 nm) and 572.205 Hz (405 nm) and the resulting signal demodulated using lock-in amplification. Both LEDs were connected to a Doric Mini Cube filter set (FMC5_E1(405)_E2(460–490)_F1(500–550)_S) and the excitation light was directed to the animal via a mono fiber optic patch cord (DORIC MFP_400/460/900–0.48_2m_FC/MF2.5). The power of the LEDs was adjusted to be 30 µW at the end of the patch cord. The resulting signal was detected with a photoreceiver (NewPort model 2151). Fluorescent signal data was processed in real-time and acquired at a sampling rate of 100 Hz. Data was then exported to MATLAB (Mathworks) for offline analysis using custom-written scripts to downsample the signals to ~10 Hz for analysis. The 465/470 nm and 405 nm data were first individually fit with a second order curve which was then subtracted to remove any artifacts due to bleaching, then the z-score was calculated using the standard deviation and mean signal recorded during baseline. Approximately 3 min of data for each condition (homecage and novel environment) were used for analysis. As previously reported, GCaMP fluorescence increased in response to a novel environment^47^. However, increases in bulk calcium level do not provide insight into the underlying dynamics of the system. That is because mean values are insensitive to the spatio-temporal pattern of the signal which could arise from changes in correlated neuronal ensemble firing patterns or through changes in gain via persistent intracellular calcium release^48–50^. Here we asked if LoTRA could be used to identify patterns embedded within signals between conditions as unlike persistent intracellular calcium release, recruitment of underlying neural ensembles would be expected to significantly alter the pattern embedded within the population-level signal. To this end, delay time *t* (range 10–100) and the embedding dimension *m* (range 2–5) for phase-space reconstruction of fiber photometry signals were determined the same as described above. Similar to other non-periodic physiological data, the threshold was set to generate a recurrence rate around 2%^24^. Re-running the analysis with a recurrence rate on the order of 5% gave similar results (not shown). For miniature microscopy data, activity of PVN CRH neurons from *n* = 4 mice were recorded continuously for at least 15 min at 20 FPS, 40% LED power and 2.5 gain, using the nVista Acquisition Software (Inscopix). The video files were cropped with ImageJ, preprocessed by Min1pipe^51^, and analyzed with custom-scripts in MATLAB. The data corresponded to the same conditions (i.e., homecage prior to novel environment, novel environment, back to homecage) used for fiber photometry analysis.

### Machine-learning model

The distance matrix from the scaled 3-dimensional Dynamic Spiral drawing time-series data were transformed into a local topological matrix with which the high curvature 8-bit binary codes (64–191) were used to generate a binary sparse matrix. Recurring topological patterns were then extracted by recurrence statistics^13^ and fed into a support vector machine. A nested leave-one-out cross validation procedure was used to evaluate hyperparameter-tuned model performance.

### Data and statistical analysis

Gait and in vivo recurrent pattern signal complexity was measured by recurrence entropy. It is based on the point density-based measure, recurrence rate (RR):

7 $$RR = \frac{1}{{N^2}}\mathop {\sum }\limits_{i,j = 1}^N R_{i,j}$$

which is a measure of the density of non-zero elements in the binary matrix^1,13^. The probability of finding a diagonal line of exact length $l$ in the binary matrix is given by:

8 $$p\left( l \right) = P(l)/N_l$$

where $P\left( l \right)$ represents the histogram of diagonal lines of length $l$, and $N_l$ is the total number of diagonal lines. Entropy (ENT), which is a measure of signal complexity, is the Shannon entropy of the probability $p\left( l \right)$:

9 $${\rm{ENT}} = - \mathop {\sum }\limits_{l = l_{{\rm{min}}}}^N p\left( l \right)ln\;p(l)$$

where $l_{{\rm{min}}}$ is commonly set to 2 as it takes a minimum of two points to define any line^1,23^. All analyses were done in Prism, SPSS, and R (R: A language and environment for statistical computing. R Foundation for Statistical Computing, Vienna, Austria). Statistical analysis used two-tailed t-tests or analysis of variance, and α was set at 0.05.

### Reporting summary

Further information on research design is available in the Nature Research Reporting Summary linked to this article.

## Supplementary information

Supplementary Information

Reporting summary

## Data Availability

The datasets generated during and/or analyzed during the current study are either publicly available or available from the corresponding authors on reasonable request.

## Code availability

R software (R Core Team (2018). R: A language and environment for statistical computing. R Foundation for Statistical Computing, Vienna, Austria) and associated source code and packages (e.g., deSolve, apcluster, nonlinearTseries, TSPred, crqa, tseriesChaos, e1071, wvtool, stats, plot3D) are freely available (https://www.R-project.org/). The custom-scripts that were used in MATLAB are available at https://github.com/leomol/MSA.
