# Supplementary material for: A versatile computational algorithm for time-series data analysis and machine-learning models
Source: NPJ Parkinsons Dis. 2021 Nov 9;7:97. doi: 10.1038/s41531-021-00240-4 (PMC8578326; doi:10.1038/s41531-021-00240-4)
Supplement: Supplementary file 1 — Supplementary Information [file 41531_2021_240_MOESM1_ESM.pdf]

# Supplementary Table

## Supplementary Table 1. Deep learning and LoTRA handwriting models using the same dataset

| | CNN-based Model 1[a] | CNN-based Model 2[b] | CNN-based SVM Model 3[c] | CNN-based Model 4[d] | LoTRA-based SVM Model |
|---|---|---|---|---|---|
| Data used[1] | Wacom Cintiq 12WX Graphics Tablet | Wacom Cintiq 12WX Graphics Tablet | Wacom Cintiq 12WX Graphics Tablet | Wacom Cintiq 12WX Graphics Tablet | Wacom Cintiq 12WX Graphics Tablet |
| Spiral tests included | 2 (SST & DST) | 2 (SST & DST) | 1 (DST) | 2 (SST & DST) | 1 (DST) |
| Feature vector length[2] | 128 | 100 | 4096 | 128 | 9 |
| Accuracy | 79.6% | 85.5% | 94% | 96.5% | 98.6% |
| F1 score | - | 85.2% | 95% | 97.7% | 99% |

[1]Data correspond to the Parkinson Disease Spiral Drawings Using Digitized Graphics Tablet Data Set (see Methods). CNN: convolution neural network-based deep-learning models; [a]ref[9]; [b]ref[14]; [c]ref[5]; [d]ref[6]. SVM: Support Vector Machine; SST: Static Spiral Test; DST: Dynamic Spiral Test.

[2]For the CNN-based models these represent the flattened layer.

# Supplementary Figures

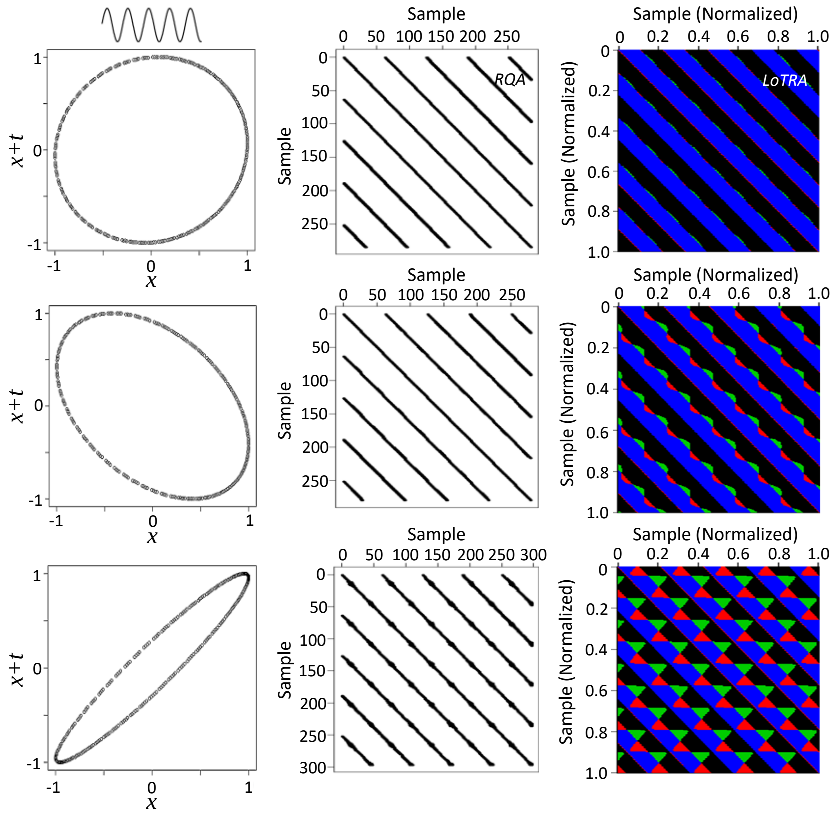


## Supplementary Figure 1. Different sine wave topological phase-space reconstructions

Three different topological phase-space reconstructions of the sine wave (shown on top) were created with increasing eccentricity. The 2-dimensional phase-space trajectory with varying time-delay ($t$) embedding values of $t$ = 15, $t$ = 20, and $t$ = 3 were used to create the three different topologies (left). Recurrence plots for each of the three phase-space reconstructions illustrating the characteristic diagonal line patterning (center). The varicosities associated with the hairpin loop-like structure recurrence plot (bottom) represent the unwanted parallel trajectory segments that are identified as recurrent points. LoTRA of the same three phase-space reconstructions (right). Blue and black ribboning correspond to the shallow curvature of the phase-space trajectory for increasing and decreasing phases of the trajectory; while the red and green pattering correspond to the increased curvature with increasing eccentricity associated with the vertices.

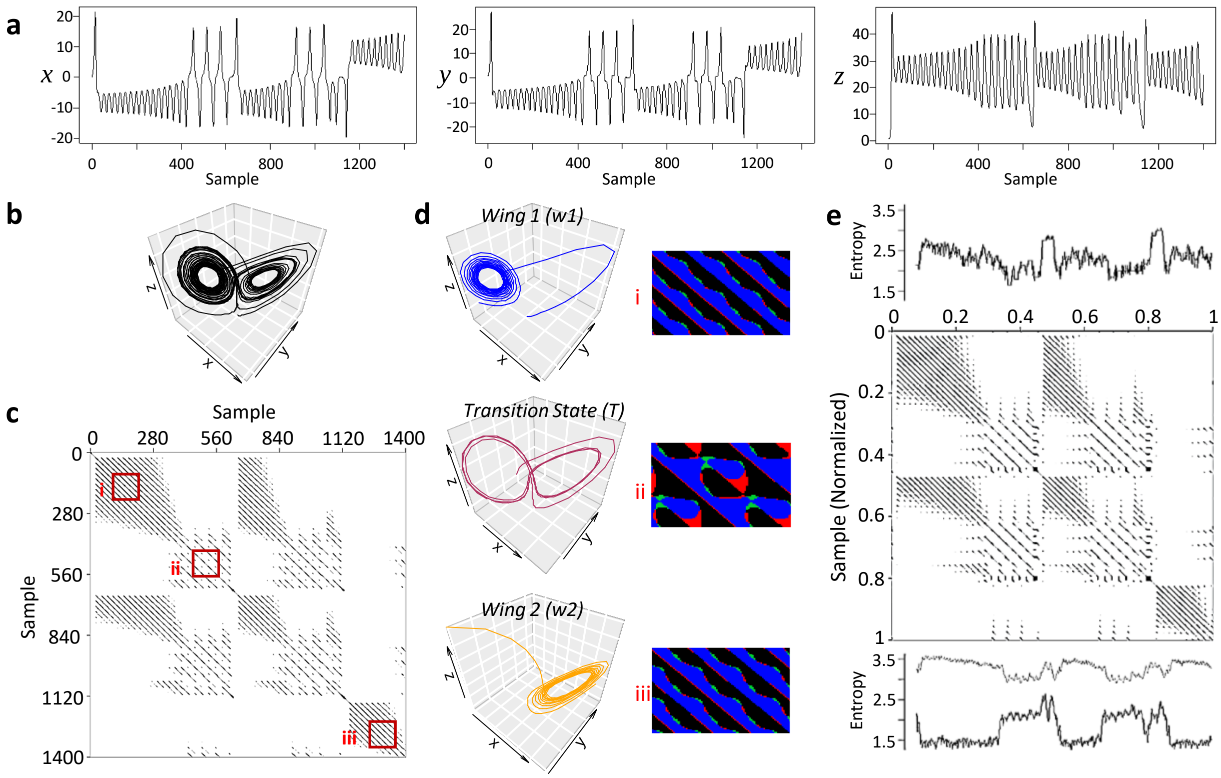


## Supplementary Figure 2. The Lorenz attractor system

a: The x, y, and z time-series of the Lorenz attractor initialized with parameters r = 28, σ = 15, and β = 8/3. b: The 3-dimensional plot of the time-series data shown in panel a. c: Recurrence plot of the Lorenz attractor system illustrating the characteristic checkerboard appearance, with “i” indicating wing 1 (w1), “ii” transition (T), and “iii” wing 2 (w2). d: 3-dimensional plots (left) of the phase-space trajectory for w1, T, and w2 with associated LoTRA patterning (right) corresponding to areas related to “i”, “ii”, and “iii” on the recurrence plot in panel c. Note the changes captured by LoTRA in both the low (black/blue) and high (red/green) curvature structures in the transition state, including the interlocking-type pattern associated with the transition state “figure-8” trajectory. e: LoTRA is also able to capture changes in topological structure complexity using a single-point sliding window (5% of the signal; 70 data points) on the Lorenz system x-coordinate time-series. Here using $m$ = 3, $t$ = 3, and $\varepsilon$ = 2 to calculate entropy for RQA (top trace) and LoTRA (bottom traces; black trace represents red/green structures, grey trace represents blue/black structures). The recurrence plot for this single channel reconstruction is shown in the middle. LoTRA can exhibit clear entropy transition states associated with the different topological structures that this system can assume.

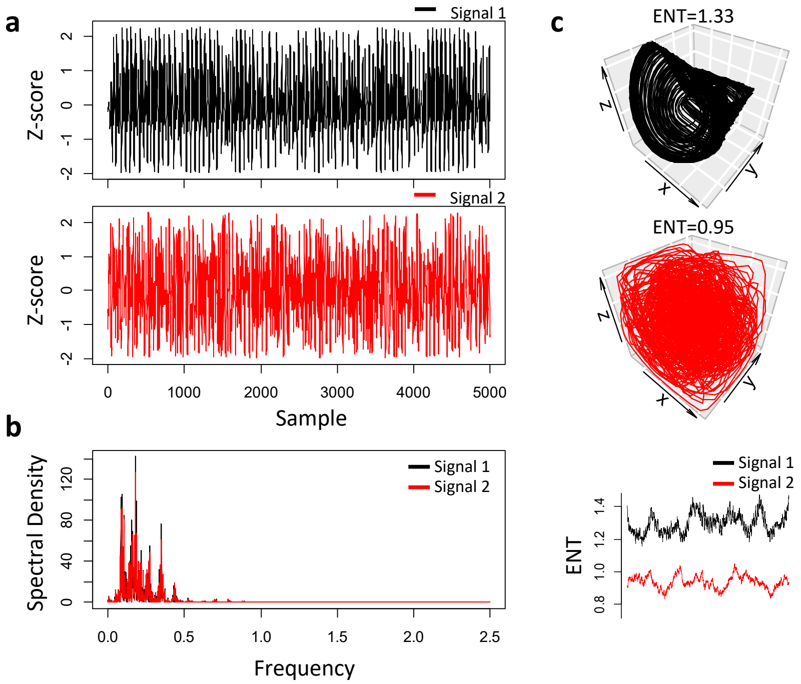


**Supplementary Figure 3. Two signals with similar spectral properties but vastly different phase-space trajectories**

a: Two signals (signal 1 and signal 2) with similar visual appearance. Signal 1 represents the x-component of the chaotic Rössler system. Signal 2 is a randomized version of signal 1 that uses a randomization process that preserves spectral features to illustrate how linear characteristics are at times insufficient to describe high dimensional nonlinear complexity[3]. b: Spectral analysis of signal 1 and signal 2 illustrating similar overlapping spectral properties. c: These same signals plotted in multi-dimensional space ($m$ = 3, $t$ = 6) reveals marked differences in the trajectory of these two signals. The different phase-space trajectories result in different topological complexity (top and middle) that can also be captured by LoTRA (ENT). Using a windowed approach (250 samples) also reveals clear separation in signal complexity between signals as the multi-dimensional signal evolves over time (bottom). The signals shown here are a modified version of those available from Goswami (2019)[3].
