# Supplementary material for: A versatile computational algorithm for time-series data analysis and machine-learning models
Source: NPJ Parkinsons Dis. 2021 Nov 9;7:97. doi: 10.1038/s41531-021-00240-4 (PMC8578326; doi:10.1038/s41531-021-00240-4)
Supplement: Supplementary file 2 — Reporting summary [file 41531_2021_240_MOESM2_ESM.pdf]

nature portfolio

Corresponding author(s): NPJPARKD-00821

Last updated by author(s): Jul 13, 2021

Reporting Summary

Nature Portfolio wishes to improve the reproducibility of the work that we publish. This form provides structure for consistency and transparency in reporting. For further information on Nature Portfolio policies, see our Editorial Policies and the Editorial Policy Checklist.

Statistics

For all statistical analyses, confirm that the following items are present in the figure legend, table legend, main text, or Methods section.

n/a	Confirmed	
☐	☒	The exact sample size (*n*) for each experimental group/condition, given as a discrete number and unit of measurement
☐	☒	A statement on whether measurements were taken from distinct samples or whether the same sample was measured repeatedly
☐	☒	The statistical test(s) used AND whether they are one- or two-sided<br>*Only common tests should be described solely by name; describe more complex techniques in the Methods section.*
☒	☐	A description of all covariates tested
☐	☒	A description of any assumptions or corrections, such as tests of normality and adjustment for multiple comparisons
☐	☒	A full description of the statistical parameters including central tendency (e.g. means) or other basic estimates (e.g. regression coefficient) AND variation (e.g. standard deviation) or associated estimates of uncertainty (e.g. confidence intervals)
☐	☒	For null hypothesis testing, the test statistic (e.g. *F*, *t*, *r*) with confidence intervals, effect sizes, degrees of freedom and *P* value noted<br>*Give P values as exact values whenever suitable.*
☒	☐	For Bayesian analysis, information on the choice of priors and Markov chain Monte Carlo settings
☒	☐	For hierarchical and complex designs, identification of the appropriate level for tests and full reporting of outcomes
☒	☐	Estimates of effect sizes (e.g. Cohen's *d*, Pearson's *r*), indicating how they were calculated

Our web collection on statistics for biologists contains articles on many of the points above.

Software and code

Policy information about availability of computer code

Data collection	Custom-scripts that were used in MATLAB are available at https://github.com/leomol/MSA.
Data analysis	R software (R Core Team (2018). R: A language and environment for statistical computing. R Foundation for Statistical Computing, Vienna, Austria) and associated source code and packages (e.g., deSolve, apcluster, nonlinearTseries, TSPred, crqa, tseriesChaos, e1071, wvtool, stats, plot3D) are freely available (https://www.R-project.org/). The custom-scripts that were used in MATLAB are available at https://github.com/leomol/MSA.

For manuscripts utilizing custom algorithms or software that are central to the research but not yet described in published literature, software must be made available to editors and reviewers. We strongly encourage code deposition in a community repository (e.g. GitHub). See the Nature Portfolio guidelines for submitting code & software for further information.

Data

Policy information about availability of data

All manuscripts must include a data availability statement. This statement should provide the following information, where applicable:

- Accession codes, unique identifiers, or web links for publicly available datasets
- A description of any restrictions on data availability
- For clinical datasets or third party data, please ensure that the statement adheres to our policy

Data are available from the corresponding authors on reasonable request if not already publicly available

Field-specific reporting

Please select the one below that is the best fit for your research. If you are not sure, read the appropriate sections before making your selection.

☒ Life sciences ☐ Behavioural & social sciences ☐ Ecological, evolutionary & environmental sciences

For a reference copy of the document with all sections, see nature.com/documents/nr-reporting-summary-flat.pdf

Life sciences study design

All studies must disclose on these points even when the disclosure is negative.

Sample size	This was a methods/computational study. However, the minimum sample size required for robust parametric-test parameter estimates (doi:10.1007/s10459-010-9222-y) was used to explore the power of our approach in comparative analyses where applicable.
Data exclusions	Not applicable.
Replication	Our experimental sample sizes were sufficient to obtain statistical power.
Randomization	This study did not require randomization.
Blinding	This study did not require blinding.

Reporting for specific materials, systems and methods

We require information from authors about some types of materials, experimental systems and methods used in many studies. Here, indicate whether each material, system or method listed is relevant to your study. If you are not sure if a list item applies to your research, read the appropriate section before selecting a response.

Materials & experimental systems

n/a	Involved in the study	
☒	☐	Antibodies
☒	☐	Eukaryotic cell lines
☒	☐	Palaeontology and archaeology
☐	☒	Animals and other organisms
☐	☒	Human research participants
☒	☐	Clinical data
☒	☐	Dual use research of concern

Methods

n/a	Involved in the study	
☒	☐	ChIP-seq
☒	☐	Flow cytometry
☒	☐	MRI-based neuroimaging

Animals and other organisms

Policy information about studies involving animals; ARRIVE guidelines recommended for reporting animal research

Laboratory animals	Crh-IRES-Cre (B6(Cg)-Crhtm1(cre)Zjh/J; stock number 012704) mice crossed with Ai148 (Ai148(TIT2L-GC6f-ICL-tTA2)-D; stock number 030328) animals were utilized. Mice were obtained from Jackson Laboratories (also see Methods).
Wild animals	Not applicable.
Field-collected samples	Not applicable.
Ethics oversight	Animal protocols were approved by the University of Calgary Animal Care and Use Committee.

Note that full information on the approval of the study protocol must also be provided in the manuscript.

Human research participants

Policy information about studies involving human research participants

Population characteristics	No new human data were collected for this study as data are related to previous studies. Details for the Parkinson Disease Spiral Drawings Using Digitized Graphics Tablet Data Set are publicly available at the UC Irvine Machine Learning Repository (https://archive.ics.uci.edu/ml/index.php). Human gait data were previously collected using the Ambulosono wearable sensor system from individuals diagnosed with mild to moderate PD with gait disturbances, and from neuro-typical individuals without any neurological or gait impairments (see Methods).

Recruitment	Not applicable.
Ethics oversight	Human data used in this study were previous collected with ethics approval obtained.

Note that full information on the approval of the study protocol must also be provided in the manuscript.
